# Comparative Transcriptomic and Proteomic Analysis to Deeply Investigate the Role of Hydrogen Cyanamide in Grape Bud Dormancy

**DOI:** 10.3390/ijms20143528

**Published:** 2019-07-18

**Authors:** Muhammad Khalil-Ur-Rehman, Wu Wang, Yang Dong, Muhammad Faheem, Yanshuai Xu, Zhihong Gao, Zhen Guo Shen, Jianmin Tao

**Affiliations:** 1 College of Horticulture, Nanjing Agricultural University, Nanjing 210095, China; 2 College of Life Sciences, Nanjing Agricultural University, Nanjing 210095, China; 3 The State Key Laboratory of Crop Genetics and Germplasm Enhancement, Nanjing Agricultural University, Nanjing 210095, China

**Keywords:** hydrogen cyanamide, dormancy, proteomic, transcriptomic, grape, DEGs

## Abstract

Hydrogen cyanamide (HC) is an agrochemical compound that is frequently used to break bud dormancy in grapevines grown under mild winter conditions globally. The present study was carried out to provide an in-depth understanding of the molecular mechanism associated with HC releasing bud dormancy in grapevines. For this purpose, RNA-seq based transcriptomic and tandem mass tag (TMT)-based proteomic information was acquired and critically analyzed. The combined results of transcriptomic and proteomic analysis were utilized to demonstrate differential expression pattern of genes at the translational and transcriptional levels. The outcome of the proteomic analysis revealed that a total of 7135 proteins (*p*-value ≤ 0.05; fold change ≥ 1.5) between the treatments (HC treated versus control) were identified, out of which 6224 were quantified. Among these differentially expressed proteins (DEPs), the majority of these proteins were related to heat shock, oxidoreductase activity, and energy metabolism. Metabolic, ribosomal, and hormonal signaling pathways were found to be significantly enriched at both the transcriptional and translational levels. It was illustrated that genes associated with metabolic and oxidoreductase activity were mainly involved in the regulation of bud dormancy at the transcriptomic and proteomic levels. The current work furnishes a new track to decipher the molecular mechanism of bud dormancy after HC treatment in grapes. Functional characterization of key genes and proteins will be informative in exactly pinpointing the crosstalk between transcription and translation in the release of bud dormancy after HC application.

## 1. Introduction

Dormancy is a developmental phase that normally occurs in deciduous plants to endure harsh environmental conditions during the winter season. The length of exposure to low temperatures is usually employed to define the chilling hours required for different tree species to regulate dormancy [1,2]. Inadequate exposure of plants to chilling hours leads to several disorders such as partial anthesis, low shoot vigor, hindered or uneven bud break, and poor flower development [3]. Therefore, it is indispensable to understand the molecular mechanism of bud dormancy release in the grape (*Vitis vinifera* L.) to increase the economic yield in mild winter regions. 

Grapes are one of the most important deciduous fruit crops widely cultivated around the globe. Table grapes are considered highly nutritious as they can be consumed fresh or in a processed form [4,5]. According to the International Organization of Vine and Wine (OIV) report, global table grape production had shown a stable growth trend due to prompt viticulture growth in China [4,6]. However, in southern China, it is challenging to achieve a consistent fruit crop every year under sheltered cultivation systems owing to insufficient chilling hours. It is quite clear that variable and uneven bud break remained notable obstacles in attaining consistent high yields and good quality fruits in many fruit plants including kiwifruit, cherries, peaches, and grapes [7]. In the meantime, insufficient chilling hours are also considered as a significant contributing factor for vine yield losses. 

To artificially compensate for the chilling requirements, various chemicals have been applied by growers to overcome the problem of insufficient chilling. Among these chemicals, potassium nitrate (KNO3), calcium cyanamide (CaCN2), and hydrogen cyanamide (HC) are the most common ones that are frequently used for breaking dormancy in deciduous fruit crops [3,8]. 

Hydrogen cyanamide is an effective agrochemical that is utilized for dormancy release in commercial production of grapes at larger scales [9,10]. Studies revealed that HC treatment persuaded a transient increase in hydrogen peroxide (H_2_O_2_) levels and substitutive oxidase transcripts [11,12]. Moreover, reports illustrated that HC-induced bud break is enhanced by calcium (Ca^2+^) signaling and stimulated changes in phosphorylation and transcription regulators [13]. Despite the extensive global use of HC by the growers for breaking dormancy, the underlying mechanism explaining its role in dormancy breaking is still not quite clear. Previous reports on grape bud dormancy hypothesized that increases in H_2_O_2_ and catalase inhibition were the key physiological changes observed under HC treatment [14,15,16]. Recent studies examined the transcriptional regulation of some genes including *EBB* (early bud break) in different plants before and after HC treatment [17,18]. Besides, the diverse expression pattern showed by the majority of genes in grape buds treated with HC indicated the association of hormone signaling, hypoxia, and oxidative stress with HC-stimulated dormancy release [12,19,20,21].

Proteomic and genomic reports in *Arabidopsis* verified the metabolic shift indicating the involvement of genes in fermentative and glycolysis pathways, including those most intensely persuaded by hypoxia [16]. In perennial fruit plants, a subtractive suppression hybridization (SSH) approach was used to investigate the number of genes involved in the dormancy mechanism [22]. A compendium of scientific literature investigated the dormancy mechanism using transcript profiling or microarrays in perennial plants [3]. Omics-based investigations are being used in different organisms like plants, as well as in humans [23,24]. In fruit trees, ‘omic’ technologies (e.g., proteomics, metabolomics and transcriptomics) have been extensively used to examine the underlying molecular and physiological processes during different developmental phases of the plant. 

Bud dormancy mechanisms in perennial fruits has been previously explored at the molecular level using omics-based approaches [3,5,25,26]. However, the effect of HC in the release of grape dormancy by coupling transcriptomic and proteomic approaches has not been yet investigated. In this study, we performed a unique attempt by utilizing both proteomic and transcriptomic approaches to investigate the dynamic changes occur in gene expression and protein patterns related to artificially induced bud dormancy in grapevine. The results have the potential to enhance table grape production in mild winter regions by providing an in-depth understanding of the mechanism involved in bud dormancy regulation.

## 2. Results

### 2.1. HC Application and its Effect on Bud Break

**Figure 1:**
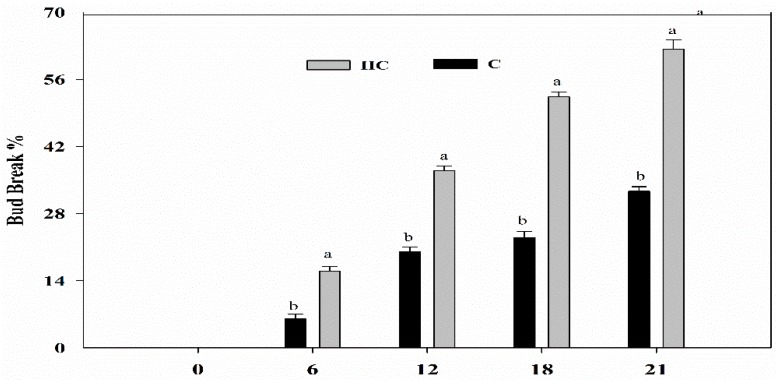
Effect of hydrogen cyanamide (HC) application on percentage of grape bud break. Single node cuttings were prepared and placed in 500 mL glass jars filled with water and percentage of bud break was assessed at 6, 12, 18 and 21 d. Vertical lines above the means bars indicate standard error (*n* = 3; *p* < 0.05) using Turkey’s HSD post hoc test. a–b represents significant difference between treatments.

Under forced conditions, the grape buds were treated with HC to check its effect on the release of grape bud dormancy. HC application stimulated the bud break in grapes. After HC treatment on 20th February 2017, the bud break percentage after 18 days of treatment reached 64%, which designated that the buds had released their dormancy. However, when treated with water (C) the bud break was observed to be only 23% at the same interval of 18 days. This clearly showed that the rate of bud break after HC treatment was higher than after water treatment (Figure 1). 

### 2.2. Transcriptomic Analysis Overview 

**Figure 2:**
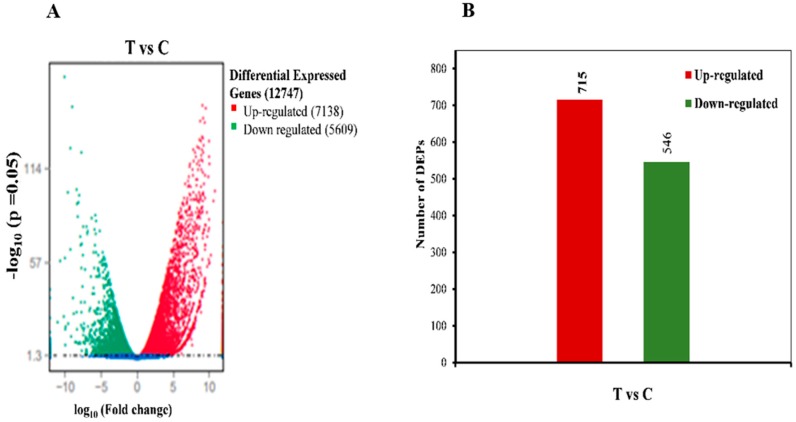
(**A**) Expression pattern of differentially expressed genes in treated and control samples. The differential genes are shown in red and green colors. (**B**) Quantification of differentially expressed proteins in treated (T) and controlled bud samples. Number of up- and down-regulated were represented in colored bars. A quantitative ratio over 2 was considered up-regulation while a quantitative ratio less than 0.5 was considered as down-regulation (*p* < 0.05).

Before and after treatment of bud samples with HC, RNA-seq analysis was carried out, where each sample was comprised of two biological replicates. For each sample, approximately 85–87% high-quality reads were mapped to the grape reference genome. (Table S1). A total of 30,867 transcripts were obtained from four samples. Based on *p*-value (*p* < 0.05) the differentially expressed genes (DEGs) were screened with a false discovery rate (FDR) of ≤0.001 and absolute fold change value of |log2ratio|≥1. A sum of 12,747 differentially expressed genes was found in T/C comparison including 7138 genes with higher expression level while 5609 genes with lower expression level were observed respectively (Figure 2A, Table S2).

### 2.3. Dynamic Profiling of Proteome after HC Treatment of Grape Buds/Detection of Proteins in Treated and Control Samples

In this study, a quantitative proteomic analysis was carried out between treated and control samples to understand the molecular mechanism. The total proteins in the T/C samples were labeled with tandem mass tag (TMT) using UPLC-MS/MS systems. The variation in the relative abundance of any given protein was determined on the basis of TMT 6-plex reporter ion ratios. To minimize peptide identification error, the matching error of the databank search approach to <2 ppm was used. Only those proteins which showed >1.5-fold change in relative quantity and *p* < 0.05 were designated as differentially expressed proteins (DEPs). A total of 7135 DEPs were identified among which 6224 proteins were computed with assessed false discovery rate (FDR) 1%. The results specified that most of the proteins showed up-regulation (715) while fewer proteins showed down-regulation (546) in T/C comparison (Figure 2B). 

### 2.4. Comparative Analysis of Proteomic and Transcriptomic Results

**Figure 3:**
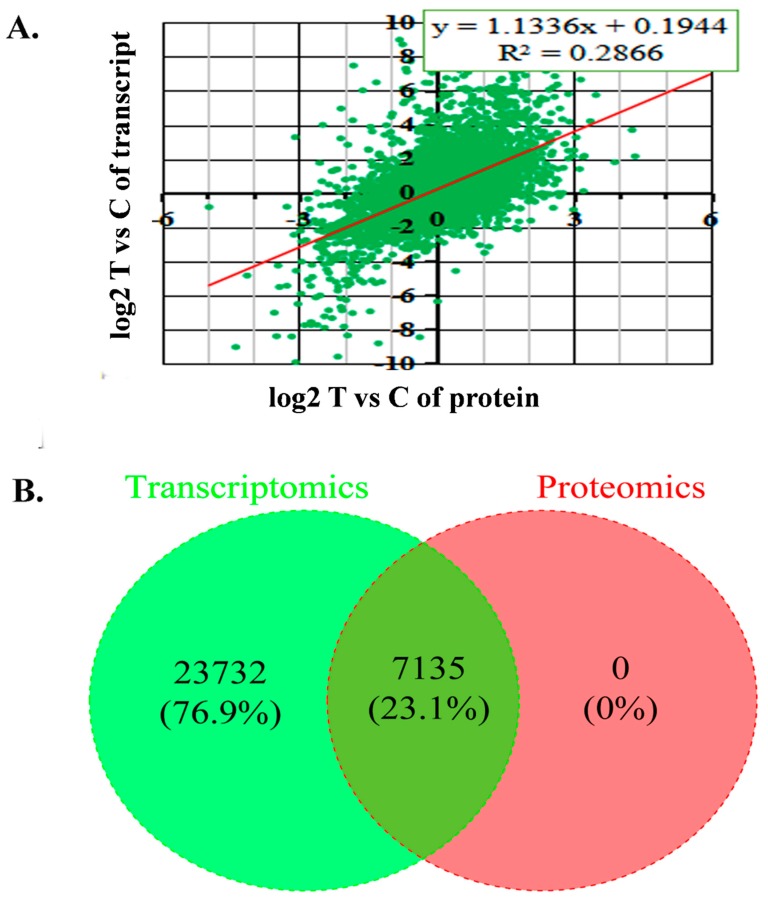
Correlation analysis between differentially expressed genes and proteins in treated and control samples. (**A**) The *x*-axis illustrates the expression pattern of the differentially expressed proteins (DEPs) and the *y*-axis illustrates the expression pattern of the differentially expressed genes (DEGs) in treated and control samples. (**B**) Venn diagram shows the quantity co-regulated proteins and genes in the treated and control bud samples.

A correlation analysis between quantitative proteomic and RNA-seq data was carried out. The expression patterns of all quantified proteins and their related transcripts in T/C samples indicated a lower correlation. The expression ratio of mRNAs and their related proteins with similar trends were designed, and positive correlation was quantified between DEGs and DEPs (Figure 3). 

RNA-seq analysis identified 12,747 DEGs (T/C), and 1261 (T/C) DEPs contained quantifiable information with respect to their samples/treatments. Exactly 7135 genes designated as co-regulated DEPs-DEGs, containing 13 (T/C) genes regulated at the proteomic (*p* < 0.05 and >1.5-fold) and transcriptomic levels FDR <0.001 and >2-fold). A similar trend of 44 and 39 genes were observed in T/C samples among 7135 core DEP-DEG genes while 1371, 1941 and 2562 genes showed a contrasting trend in T/C samples. We proposed that some of these genes might be involved in grape bud dormancy regulation (Figure S1). 

### 2.5. Enrichment Analysis of DEGs-DEPs Related to the GO and KEGG Pathways 

**Figure 4:**
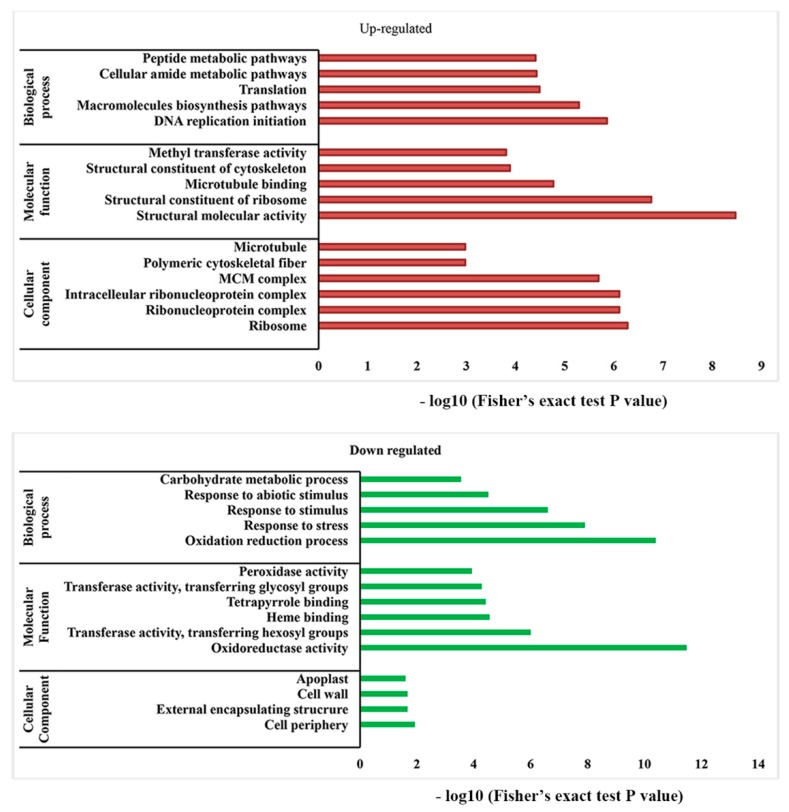
Gene ontology functional enrichment analysis of co-regulated genes and proteins in treated and control samples.

In the present study, 7135 co-regulated DEGs-DEPs genes had gene ontology (GO) annotations. GO terms were assigned to 7135 core DEGs and DEPs to perform a functional analysis. The results comprised of the main GO terms of biological processes, cellular components and molecular functions, including essential functional groups (Figure 4). In “biological process”, the largest categories found were “oxidation-reduction process” and “response to salt stress”. The genes/proteins associated with these categories had lower expressions. In “cellular component” the main categories found were “ribosome” and “ribonucleoprotein complex”. The proteins/genes associated with these categories were up-regulated. For “molecular function” the major categories found were “structural molecular activity” and “structural constituent of ribosome”. The proteins/genes associated with these categories were up-regulated.

Based on the Kyoto Encyclopedia of Genes and Genomes (KEGG) analysis, the main identified DEPs-DEGs (*p*-value ≤ 0.05) were annotated to 14 enriched KEGG pathways. The results depicted that five KEGG pathways were mostly enriched at both proteomic and transcriptomic levels that includes “starch and sucrose metabolism” (vvi00500), “pyruvate metabolism” (vvi00620) “ribosome” (vvi03010), “circadian rhythm” (vvi04712) and “phenyl propanoid biosynthesis” (vvi00940) (Figure S2). Furthermore, secondary metabolism pathways like “flavonoid biosynthesis” (vvi00941) “phenyl propanoid biosynthesis” (vvi00940) were also enriched in core DEGs-DEPs genes in the KEGG analysis. 

### 2.6. Differentially Expressed Genes Related to Metabolism and Hormone Signaling 

In this study, 1941 genes were identified in treated and control bud samples that were related to metabolism. Among of those, 713 were up-regulated and 346 were down-regulated in T/C samples. In the hormone signaling pathway, 276 genes were found to be differentially expressed in T/C samples. ABA and GA signaling pathways were further examined. In the ABA signaling pathway, five genes annotated as PYR/PYL, of which three genes were up-regulated while two genes were down-regulated in T/C samples. Eight genes related to protein phosphatase 2 C (PP2C) family showed lower expression, while one gene related to Snrk2 family showed a higher expression level in T/C samples. In the GA signaling pathway, two genes related to DELLA family showed elevated expression level while genes related to GID1 and GID2 family showed lower expression level in T/C samples (Table 1). 

### 2.7. Validation of RNA-seq Results

To verify the RNA-seq results, an RT-qPCR assay was carried out where nine selected genes had shown differential expression patterns before and after HC treatment. The randomly selected genes encoded with different proteins are mentioned: Lipoxygenase (VIT_01s0010g02750), Glutathione peroxidase (VIT_02s0025g03600), Glutamine synthetase (VIT_17s0000g01910), Glutathione S-transferase (VIT_12s0028g00920), Cytochrome c oxidase subunit 2 (VIT_00s0438g00010), Ubiquitin carboxyl-terminal hydrolase (VIT_06s0009g00960), zinc ion binding (VIT_01s0146g00060), Serine/threonine-protein kinase (VIT_04s0008g05500), oxidoreductase (VIT_00s0531g00040). The RT-qPCR and RNA-seq results were highly correlated (Figure S3). 

### 2.8. Expression Pattern of DEGs-DEPs in Cluster Analysis

**Figure 5:**
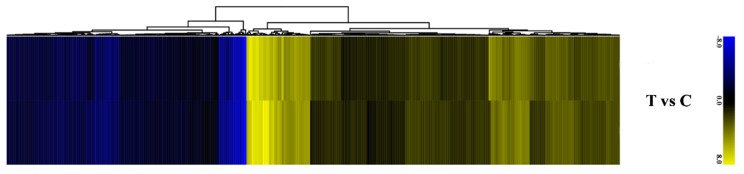
Hierarchical clustering analysis of DEGs and DEPs based on transcriptomic and proteomic data. The heat map is linked by a dendrogram representing a clustering of transcriptomic data or proteomic data (left side). The color code is as follows: blue indicates down-regulated transcripts or proteins; yellow indicates up-regulated transcripts or proteins; black indicates unchanged transcripts or proteins. Each row represents the log2 (treated/C) of a gene or protein. The color scale of the heat map ranges from saturated blue (value, −8.0) to saturated yellow (value, 8.0) in the natural logarithmic scale.

The identified 7135 co-regulated DEGs and DEPs were divided into three groups on the basis of their expression pattern in T/C bud samples at the transcriptomic and proteomic levels. The genes (518) in group A were up-regulated in T/C buds and categorized as up-up-regulated genes. The pattern of genes (371) expression in group B were down-down-regulated in both samples and categorized as down-down-regulated genes; the genes (2562) that did not show any change in expression pattern were categorized as unchanged-unchanged group C (Figure 5). 

## 3. Discussion

Dormancy plays a significant role in undergoing the unfavorable environmental conditions of the winter season in perennial fruit plants [27]. Various biochemical, molecular and physiological activities are associated with the transition of dormancy to initiate bud growth [28]. Recent reports about proteomic and transcriptional analysis revealed that energy metabolism is an obligation for dormancy release, including in the development of leaves and flowers. Meristem requires adequate energy from the primary tissue to maintain bud growth at bud break time [29,30]. TMT-based proteomic and RNA-seq based transcriptomic analysis was performed to measure the transcript and protein expression in grape buds before and after treatment with HC in this study. Results revealed that five proteins including a TPR-like superfamily protein, triosephosphate isomerase, putative 2, 3-bisphosphoglycerate-independent phosphoglycerate mutase, and ribulose bisphosphate carboxylase small chain were involved in energy metabolism that is associated with the dormancy release of treated and control grape buds. The tricarboxylic acid cycle/glycolysis is the key metabolic pathway in plants that is linked with plant respiration. It also provides mitochondria with pyruvate which is essential for the biosynthesis of various metabolic compounds. Energy metabolism is one of the key factors connected with the release of bud dormancy, as demonstrated in our study. Various genes related to energy metabolism like triosephosphate isomerase, chloroplastic, Malate dehydrogenase [NADP], chloroplastic, Ribulose bisphosphate carboxylase small chain and chloroplastic-like showed differential expression patterns in treated samples compared to control samples. Thus, HC promoted the activity of proteins and genes related to energy metabolism that possibly leads to advanced bud break in grapes (Figure 1). Previous reports also revealed that in grape buds treated with HC, alcohol dehydrogenase was induced under respiratory stress. Respiratory hindrance and changes in the ATP:AMP ratio were also noted to be associated with dormancy release [31]. Thus, HC promoted the action of proteins and genes linked with energy metabolism which might play a key role in earlier dormancy release in grapes.

Many proteins play important roles in various metabolic processes. Previous report suggested that higher expression of eukaryotic initiation factor proteins are associated with dormancy release in *Pinus sylvestris* L [29]. In the current study, a protein with accession number (VIT_17s0000g06670.t01), identified as eukaryotic translation initiation factor 5A-2 (ETIF-5A-2) (Table 2), showed high expression in HC treated grape buds. Consequently, in the associated mechanism for dormancy release, eukaryotic initiation factor proteins might be essential for their involvement in cell division and protein synthesis during HC treatment. As a component of protein metabolism, elongation factor (EF)-1 plays a crucial role in cell division as well as in protein synthesis in meristematic tissues [32]. In beech seed dormancy, EFs might play a key role in dormancy release, cell division and protein metabolism of root meristem [33]. EF TuB, chloroplastic, ETIF-5A-2 and peroxidases found in core DEPs and DEGs designated that they might play a key role in dormancy release after HC treatment. 

**Figure 6:**
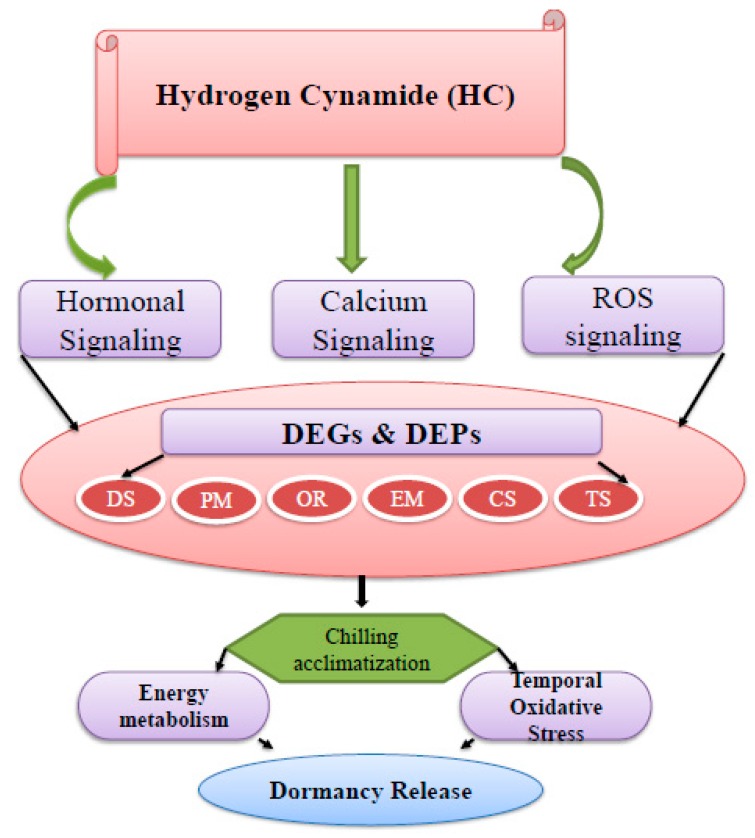
Proposed molecular model of bud dormancy release in grapes after HC application. In this model after HC application, hormone signaling, ROS signaling and Ca^+2^ signaling control the expression patterns of different type of genes and proteins including DS (defense and stress) PM (protein metabolism), OR (oxidation-reduction), EM (energy metabolism), CS (cell structure) and TS (transcription and signaling).

Oxidative stress is an imperative component of the dormancy release process. Reports stated that antioxidants system involving ascorbate peroxidase, peroxidase superfamily and superoxide dismutase is considered essential for dormancy release [29,31,33]. Table 2 revealed that proteins related to oxidoreductase including ascorbate peroxidase (VIT_08s0040g03150.t01) and superoxide dismutase (VIT_10s0042g00100.t01) showed differential expression patterns. The protein with accession no: VIT_08s0040g03150.t01 displayed lower expression in the HC-treated grape buds while protein with accession no: VIT_10s0042g00100.t01 exhibited higher expression during dormancy release. Peroxidase is involved in the generation of H_2_O_2_ by oxidation of NADH and showed higher expression in peach buds. Ascorbate reduced peroxidase activity was seen as a signaling cascade during dormancy release in the Japanese Apricot [3]. Previous reports illustrated that elevated level of antioxidant activity corresponded with bud dormancy release [3,34]. In agreement with it, the majority of the proteins allied to oxidoreductase activity showed elevated expression patterns in HC-treated buds during dormancy release, apart from ascorbate peroxidase which showed no expression (Table 2). A temporarily elevated level of H_2_O_2_ was leading toward endodormancy release in the grape bud after application of the catalase inhibitor HC [15]. In HC-treated grape buds, a transitory up-regulation of H_2_O_2_ led towards dormancy release and acted as a transitory signal from the rest period to bud break [34]. The rising signal indicated that oxidative stress may be involved in bud dormancy release in deciduous fruit plants [2,35]. The reduced H_2_O_2_ level is catalyzed by peroxidases and is recognized as cell wall loosening enzymes. It may help in the modulation of reactive oxygen species (ROS) synthesis that is associated with cell walls during the bud break of dormant grape buds after HC application [35]. In *Arabidopsis*, ROS production occurred through NADPH oxidase that is stimulated by the expansion of cell walls via the triggering of Ca^2+^ channels [36]. Our results revealed that proteins related to oxidoreductase activity were differentially expressed in HC-treated grape buds. The protein with accession no: VIT_08s0040g03150.t01 had a lower expression while the protein with accession no: VIT_10s0042g00100.t01 showed higher expression in HC-treated grape buds. In the database of DEGs, 101 identified genes were correlated with oxidoreductase activity (Figure 6). Thus, we can speculate that application of HC was possibly intricated toward the advancement of oxidative stress and consequent to earlier dormancy release in HC-treated buds compared with control buds. 

In *Arabidopsis*, a proteomic study revealed that a cytoskeleton element alpha-2, 4-tubulin seems to rely on GA action in germination. Recent studies observed that tubulin-A showed up-regulation during winter dormancy in tea plants while tubulin alpha-2 chain exhibited higher expression during fall dormancy and lower expression at dormancy release [30,37]. According to our findings, predicted: tubulin alpha chain, partial (VIT_18s0001g08250.t01) and predicted: tubulin alpha-3 chain (VIT_03s0088g00380.t01) showed up-regulation in HC-treated buds and no significant change was observed in untreated samples. As a result, the application of HC might stimulate the cell elongation as well as cell division that facilitates bud dormancy release in grape. Studies were carried out to determine the role of Ca^2+^ signaling in grape bud break. A previous study proposed that Ca^2+^ signaling plays an important role in grape bud break. A key protein named calreticulin related to calcium signaling by altering calcium homeostasis during dormancy regulation [3]. Calreticulin is considered a key protein in the GA signaling pathway owing to its significance in hormonal signaling cascades, which in turn might be involved in dormancy release of beech seed and its germination [33]. Hormones are considered key elements in dormancy regulation based on the investigation of expression patterns of hormonal signaling genes to illuminate the dormancy mechanism in perennial fruit plants [38]. The genes related to the *ABRE* and *PP2C* families are considered as ABA receptors in grapevine buds and are controlled at the transcriptional level [10]. The elevated expression level of genes related to the *PYR/PYL* family and decreased levels in *PP2C* family genes were observed in HC-treated grape buds. Genes related to the *DELLA* family exhibited higher expression while genes related to the *GID1* and *GID2* families exhibited lower expression in control and treated samples. The calreticulin protein and its related genes showed down-regulation after HC application in the current report, whereas no significant difference was observed in untreated buds. Consequently, HC application may have the potential influence the release of bud dormancy in grapevines. 

## 4. Material and Methods

### 4.1. Plant Materials

A table grape cultivar ‘Shine Muscat’ (*V. vinifera L× Vitis labruscana Bailey*) was used as the research material in this study. The grapevine plants were six years old that were raised under sheltered tunnels located at the experimental vineyard of Nanjing Agricultural University, Nanjing, P. R. China. Bud samples were excised from one-year-old canes grapevines on 20 February 2017, and transported to the laboratory [10,39]. Single node cuttings were prepared from each cane and mixed. Each treatment group had ten single node cuttings randomly drawn from the mixture. The basal part of the cuttings were placed in glass bottles filled with water. The cuttings were divided into two groups: the HC-treated (T) group and control (C) group. The cuttings in the T group were treated with HC @ 5% *v/v* (‘Dormex’ SKW, Trostberg, Germany) whereas cuttings of the C group were only subjected to a distilled water treatment. Cuttings of both groups were kept at 24 ± 1 °C under a 16:8 h dark:light photoperiod at 75% humidity. The single node cuttings were placed in a growth chamber for 21 days to observe the percentage of bud break. The methodology was followed as described by [3] with some modifications. The visibility of green tissue below the bud scale was considered as bud break [12]. At each time point, 150 buds were measured from each treatment for assessment of bud break. Three biological replicates were used and samples collected from three selected plants. Each plant was designated as a biological replicate. The buds collected at 0 d from control and treated cuttings were considered as C. Furthermore, buds collected from treated cuttings after 18 d were considered as T to investigate the proteomic and transcriptomic variations (Figure S4). 

### 4.2. Extraction of Protein

Protein was extracted and purified following the methodology proposed by [40], with some modifications. After grinding the bud samples in liquid nitrogen, about 0.8 g powdered sample was homogenized in 5 mL pre-cooled protein extraction buffer as described by [41]. The homogenate was centrifuged at 13,000× *g* at 4 °C for 20 min (min). The supernatant was separated from the bud debris and vortexed after addition of Tris-phenol (pH ≥ 8.0) followed by centrifugation, at 4 °C for 20 min. Thereafter, the upper parts of phenol were gently shifted to new tubes, and 5 volume of 0.1 M methanolic ammonium acetate (MAA) in absolute methanol was added. It was shaken well and kept at −80 °C overnight for precipitation of proteins. The precipitated proteins were then washed twice with 0.1 M acetone and MAA, respectively. The dried protein pellets were solubilized into buffers as proposed by Liu et al [40]. The protein concentration was determined with a 2-D Quant kit (Merck, China) according to the manufacturer’s instructions.

### 4.3. Protein Digestion and TMT Labeling

Protein digestion was carried out following the methodology as described by [40]. Peptides were desalted by Strata X C18 SPE column (Phenomenex, USA). After that, desalted peptides were reconstituted in 0.5 M triethylammonium bicarbonate (TEAB) and processed according to the manufacturer’s protocol for the TMT Kit (Thermo Scientific, USA). Using TMT 6-plex, the tryptic peptides (100 µg of protein) were categorized to label with 127-tag (CK-1), 128-tag (CK-2), 129-tag (T1) and 130-tag (T2). The four labeled samples from each set were pooled after examining the label assimilation following the procedure as described by [42].

### 4.4. HPLC Separation and LC-MS/MS Analysis

High-performance liquid chromatography (HPLC) was used to separate labeled peptides into fractions through Agilent 300 with C18 column (5 μm particles, 4.6 mm i.d, 250 mm length) with a gradient of 2% to 60% acetonitrile (pH 10) over 80 min, and were then joined into 18 fractions. Using vacuum centrifugation, the joined peptides were dried and dissolved in 0.1% formic acid for mass spectrometry (MS) assay. LC-MS/MS analysis was carried out using EASY-nLC 1000 UPLC system combined with Q Exactive™ Plus hybrid quadrupole-Orbitrap mass spectrometer (Thermo Scientific, USA). 

The peptides were eluted using four phases linear gradient of solvent B, (0.1% formic acid in 98% ACN) about 22% for 26 min; about 40% for 12 min; 80% for 3 min; then hold at 80% for 3 min. Using a Q Exactive™ Plus hybrid quadrupole-Orbitrap mass spectrometer (Thermo Scientific) connected with online UPLC system. A frequent flow rate was kept at 300 mL/min by an EASY-nLC 1000 UPLC system. The resultant peptides were further processed using a Q Exactive™ Plus hybrid quadrupole-Orbitrap mass spectrometer (Thermo Scientific) connected online with UPLC. The entire peptides were identified at 70,000 resolutions with MS range of 350–1800 *m*/*z* in the Orbitrap for the full scan. Above a threshold ion count of 1E4, the 20 top precursor ions in the MS survey scan were detected with 30.0-s dynamic exclusion. Ion fragments were identified at 17,500 resolutions in the Orbitrap. Automatic gain control was set as 5E4 ions to avoid the congestion of the ion trap. 

### 4.5. Database Search 

MaxQuant with unified Andromeda database (v.1.5.2.8) was used to process the produced MS/MS data. Tandem mass spectra were examined to compare transcriptome databank with mass spectrometry contaminants database and reverse decoy database. The MaxQuant software was set to allow up to two missed cleavages. In a peptide, five amino acids for least peptide length and five for the highest alteration sites; mass tolerance for fragment ions are 0.02 Da while five ppm for peptide ions; and FDR ≤ 1% for peptide-spectrum matches and protein identification. The proteins identification and quantification by at least one unique peptide were analyzed with the median ratio of its matching peptides and normalized through taking the median of all calculated proteins. TMT 6-plex was selected for quantification. In Maxquant, all other parameters were set to default values. 

### 4.6. Total RNA Extraction and Illumina Sequencing

The Foregene RNA isolation kit (Foregene, Shenzen, China) was used to isolate total RNA from frozen buds, following the manufacturer instruction. For mRNA isolation, magnetic beads with oligo were used. To get smaller fragments, the fragmentation buffer was mixed with mRNA. The cDNA was synthesized using mRNA fragments as templates. Adapters were used to link the small fragments. The ABI step one plus real-time PCR and Agilent 2100 Bioanalyzer were used in qualification and quantification of sample libraries (Figure S5). The data obtained from the Illumina sequencing were uploaded in the NCBI Sequence Read Archive (SRA) (accession number, GSE127322). 

### 4.7. Bioinformatics Analysis

The data obtained after sequencing were subjected to quality control (QC). The high quality reads thus obtained were aligned to the grape reference genome (ftp://ftp.ensemblgenomes.org/pub/release-23/plants/gtf/vitis_vinifera/) (21.06.19). The aligned data were used to estimate the reads distribution and mapping ratios. Moreover, based on DEGs-DEPs pathway analysis, gene ontology enrichment analysis, and cluster analysis were carried out.

### 4.8. Validation of RNA-Seq Data by RT-qPCR

Reverse transcript quantitative polymerase chain reaction (RT-qPCR) was carried out to determine the expression of selected candidate genes as reported previously [31] (Table S3). 

### 4.9. Transcriptomics and Proteomics-Based Correlation Analysis

Transcriptomic and proteomics-based correlation analysis involved the results of TMT-based protein and transcription analysis to evaluate the possible applicability of quantitative information between proteins and genes in HC-treated and control buds. In both libraries, the correlation analysis was carried out between DEGs and DEPs using log2 values.

### 4.10. Data Analysis 

Data were analyzed for one-way ANOVA (Duncan’s multiple range test) using SPSS Software Package (v.19.0.0.0). Principal component analysis and heat map analysis were constructed using SIMCA-P (version 11.5) and Genesis software. Fisher’s exact test was performed to examine the functional category enrichment.

## 5. Conclusions 

Comprehensively, our results proposed a key role of genes and proteins involved in oxidoreductase activity and energy metabolism after HC application in the synchronization of dormancy release in grape buds at the transcriptomic and proteomic levels. The results of our study furnish a global atlas of variation in gene expression and protein accumulation in grape bud dormancy release after HC application. This study will facilitate the elucidation of candidate genes and proteins involved in grape bud dormancy and also the understanding of the role of synthetic dormancy breakers in this mechanism.

## Figures and Tables

**Table 1:** DEGs related to hormone signaling pathway in treated and control bud samples.

Gene ID	Log2FC	Regulation	Description
**ABA pathway**			
LOC100267073	1.9481	up	abscisic acid receptor PYL4
LOC100267793	2.5272	up	abscisic acid receptor PYR1
LOC 100267287	−4.1216	down	probable protein phosphatase 2C 8
LOC100255251	−3.7464	down	probable protein phosphatase 2C 24
LOC100241147	−1.752	down	protein phosphatase 2C 16
LOC100249013	1.7144	up	Serine/threonine-protein kinase SRK2A
**GA pathway**			
LOC100249084	1.7451	up	DELLA protein GAI
LOC100249385	−1.5149	down	gibberellin receptor GID1B
LOC100245654	−2.1794	down	F-box protein GID2

**Table 2:** Identification of proteins associated with dormancy release treated with HC in grapes.

**Defense and Stress Proteins**								
Protein Description	Accession No	T/CK ratio	Regulated Type	T/CK P value	MW [KDa)	Score	Coverage (%)	Peptides
PREDICTED: 18.1 kDa class I heat shock protein [Vitis vinifera]	VIT_13s0019g02930.t01	0.048	Down	0.000195616	18.165	-2	56.2	12
PREDICTED: heat shock protein 83 [Vitis vinifera]	VIT_16s0050g01150.t01	0.19	Down	0.035201	80.867	22.85	30	23
PREDICTED: small heat shock protein, chloroplastic [Vitis vinifera]	VIT_01s0010g02290.t01	0.272	Down	0.00174202	20.738	108.38	55.5	10
PREDICTED: 17.8 kDa class I heat shock protein [Vitis vinifera]	VIT_19s0085g01050.t01	0.272	Down	0.0098646	16.446	15.659	37	5
PREDICTED: 17.3 kDa class II heat shock protein [Vitis vinifera]	VIT_04s0008g01570.t01	0.369	Down	0.0044631	18.595	17.88	40.4	7
PREDICTED: heat shock 70 kDa protein, mitochondrial [Vitis vinifera]	VIT_00s0415g00030.t01	0.178	Down	0.0032378	18.004	3.6693	11.3	2
PREDICTED: chitinase-like protein 2 [Vitis vinifera]	VIT_05s0062g01320.t01	2.243	Up	0.0199792	34.869	21.874	13	3
**Protein related to energy metabolism**								
PREDICTED: probable cinnamyl alcohol dehydrogenase 1 isoform X1 [Vitis vinifera]	VIT_02s0025g03100.t01	0.318	Down	3.3667E-06	38.412	35.323	23.4	7
PREDICTED: alcohol dehydrogenase class-3 [Vitis vinifera]	VIT_07s0005g04600.t01	0.461	Down	0.0046182	25.165	25.744	34.3	8
PREDICTED: D-3-phosphoglycerate dehydrogenase 3, chloroplastic [Vitis vinifera]	VIT_09s0018g01870.t01	3.012	Up	0.0105971	66.287	134.77	32.4	18
Ribulose-1,5-bisphosphate carboxylase/oxygenase large subunit (chloroplast) [Senna tora]	VIT_16s0013g00330.t01	0.402	Down	0.026102	13.355	32.11	37.9	5
Alcohol dehydrogenase 3 [Vitis vinifera]	VIT_18s0001g15450.t01	0.225	Down	0.000163517	46.418	125.36	32.1	13
**Protein metabolism**								
Glutathione S-transferase; Glutathione S-transferase 3	VIT_12s0028g00920.t01	0.247	Down	4.6333E-06	24.966	43.569	41.9	9
PREDICTED: eukaryotic translation initiation factor 5A-2 [Vitis vinifera]	VIT_17s0000g06670.t01	2.003	Up	0.00063534	17.413	125.57	61.6	10
PREDICTED: probable glutathione S-transferase par C [Vitis vinifera]	VIT_19s0015g02890.t01	0.236	Down	0.0184029	25.337	3.4536	42.5	12
Elongation factor 1-alpha	VIT_08s0040g02330.t01	2.126	Up	0.0182398				
RNA polymerase II transcription elongation factor DSIF/SUPT5H/SPT5 (ISS) [Ostreococcus tauri]	VIT_05s0020g04440.t01	0.493	Down	0.0024588				
**Protein related to oxidation-reduction**								
cytosolic ascorbate peroxidase [Vitis vinifera]	VIT_08s0040g03150.t01	0.217	Down	0.00114319	27.985	108.81	45.5	10
Superoxide dismutase [Cu-Zn], chloroplastic	VIT_10s0042g00100.t01	2.131	Up	0.0038222	21.71	134.08	33	6
**Cell structure related proteins**								
PREDICTED: tubulin alpha chain, partial [Vitis vinifera]	VIT_18s0001g08250.t01	9.401	Up	0.023236	49.59	303.83	48.3	19
PREDICTED: tubulin alpha-3 chain [Vitis vinifera]	VIT_03s0088g00380.t01	19.369	Up	0.011762	49.554	47.385	48.4	19
**Transcription and signaling proteins**								
PREDICTED: Calreticulin/calnexin homolog [Vitis vinifera]	VIT_01s0150g00400.t01	0.716	Down	0.0046237	61.437	91.544	30.6	18

## Supplementary Materials

Supplementary materials can be found at https://www.mdpi.com/1422-0067/20/14/3528/s1.

## Abbreviations

HC	Hydrogen cyanamide
TMT	Tandem mass tag
DEPs	Differentially expressed proteins
DEGs	Differentially expressed genes
OIV	International Organization of Vine and Wine
EBB	Early bud break
SSH	Subtractive suppression hybridization
FDR	false discovery rate
ETIF	Eukaryotic translation initiation factor
EF	Elongation factor
ROS	Reactive oxygen species
MAA	Methanolic ammonium acetate
TEAB	Triethylammonium bicarbonate
HPLC	High-performance liquid chromatography
MS	Mass spectrometry
ACN	Acetonitrile. GO: Gene ontology
KEGG	Kyoto Encyclopedia of Genes and Genomes
