# Supplementary material for: Comparative Transcriptomic and Proteomic Analysis to Deeply Investigate the Role of Hydrogen Cyanamide in Grape Bud Dormancy
Source: Int J Mol Sci. 2019 Jul 18;20(14):3528. doi: 10.3390/ijms20143528 (PMC6679053; doi:10.3390/ijms20143528)
Supplement: Supplementary file 1 [file ijms-20-03528-s001.zip › ijms-535128-supplementary/Supplementary data/Table S1.docx]

| **Table S1 Reads detail based on the RNA-seq data in control and treated samples** | | | | |
| --- | --- | --- | --- | --- |
| **Type** | **CK-1** | **CK-2** | **Treated-1** | **Treated-2** |
| Raw Reads | 48721168 | 43380248 | 42804700 | 44358972 |
| Clean Reads | 47649934 | 42424012 | 41921568 | 43453342 |
| Total Mapped (%) | 41446945(86.98%) | 37275122(87.86%) | 35862944(85.55%) | 37048870(85.26%) |
| Uniquely mapped(%) | 40591499(85.19%) | 36425264(85.86%) | 35129747 (83.8%) | 36284083 (83.5%) |
| Multiple mapped(%) | 855446 (1.8%) | 849858 (2%) | 733197 (1.75%) | 764787 (1.76%) |
