# Supplementary material for: Comparative Transcriptomic and Proteomic Analysis to Deeply Investigate the Role of Hydrogen Cyanamide in Grape Bud Dormancy
Source: Int J Mol Sci. 2019 Jul 18;20(14):3528. doi: 10.3390/ijms20143528 (PMC6679053; doi:10.3390/ijms20143528)
Supplement: Supplementary file 1 [file ijms-20-03528-s001.zip › ijms-535128-supplementary/Supplementary data/Table S3.docx]

| **Table S3 Primer Used in Real time quantitative PCR** | | | |
| --- | --- | --- | --- |
| **Gene Name** | **Gene ID** | **Forward Primer** | **Reverse Primer** |
| Lipoxygenase | VIT_01s0010g02750 | CCCAGATACCATTAAGTG | GTAATCATATCGGAGTAAGG |
| Glutathione peroxidase | VIT_02s0025g03600 | CTGAGTATCCCATCTTTG | CAGCACTTATGAGATTCC |
| Glutamine synthetase | VIT_17s0000g01910 | CCAATTCCGACCAATAAG | GCACCAATACCACAATAG |
| Glutathione S-transferase | VIT_12s0028g00920 | GTTGGACATCTATGAAGAG | CCAGGATGGTCTATTACT |
| Cytochrome c oxidase subunit 2 | VIT_00s0438g00010 | GACGAGGTAGTAGTAGAT | CACTCTATTGTCCACTTC |
| Ubiquitin carboxyl-terminal hydrolase | VIT_06s0009g00960 | GAGATGATGACCTTGTAG | CGATTAGGACGATATGTG |
| zinc ion binding | VIT_01s0146g00060 | CATCTTCAGAGGAATGTG | GTAAGTGTCTTGCTAACC |
| Serine/threonine-protein kinase | VIT_04s0008g05500 | CTTCTGGACGATAACTAC | CACTCTTCTCTGATATGG |
| oxidoreductase | VIT_00s0531g00040 | GGACAGTATCAATGGAAC | TAGCCTCTCATCACTAAC |
| Vv Actin (Reference gene) | LOC100246825 | TACAATTCCATCATGAAGTGTGATG | TAGAAGCACTTCCTGTGAACAATG |
