# Supplementary figures and images for: Comparative Transcriptomic and Proteomic Analysis to Deeply Investigate the Role of Hydrogen Cyanamide in Grape Bud Dormancy
Source: Int J Mol Sci. 2019 Jul 18;20(14):3528. doi: 10.3390/ijms20143528 (PMC6679053; doi:10.3390/ijms20143528)

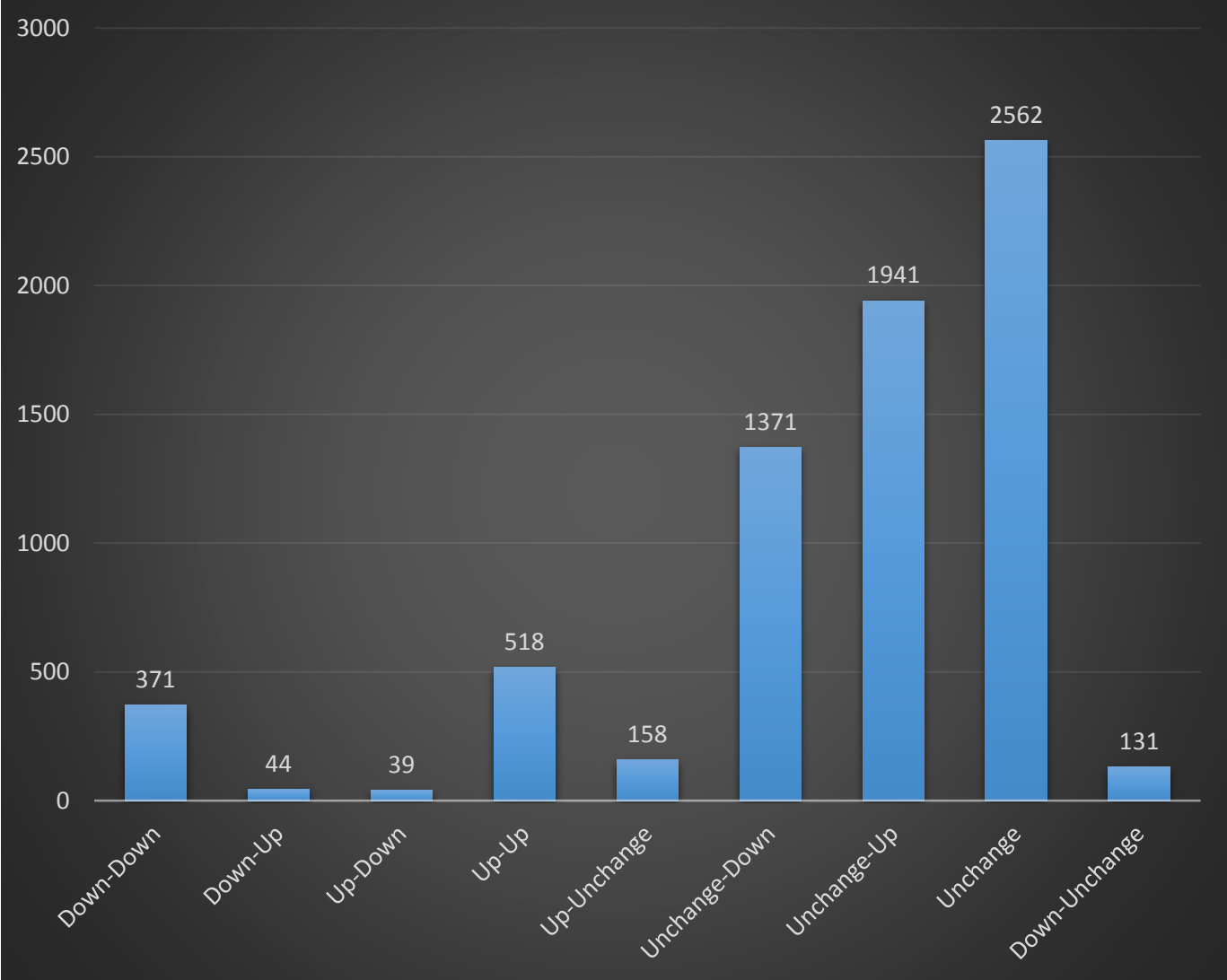

Supplement: Supplementary file 1 [file ijms-20-03528-s001.zip › ijms-535128-supplementary/Supplementary data/Figure S1.pdf]

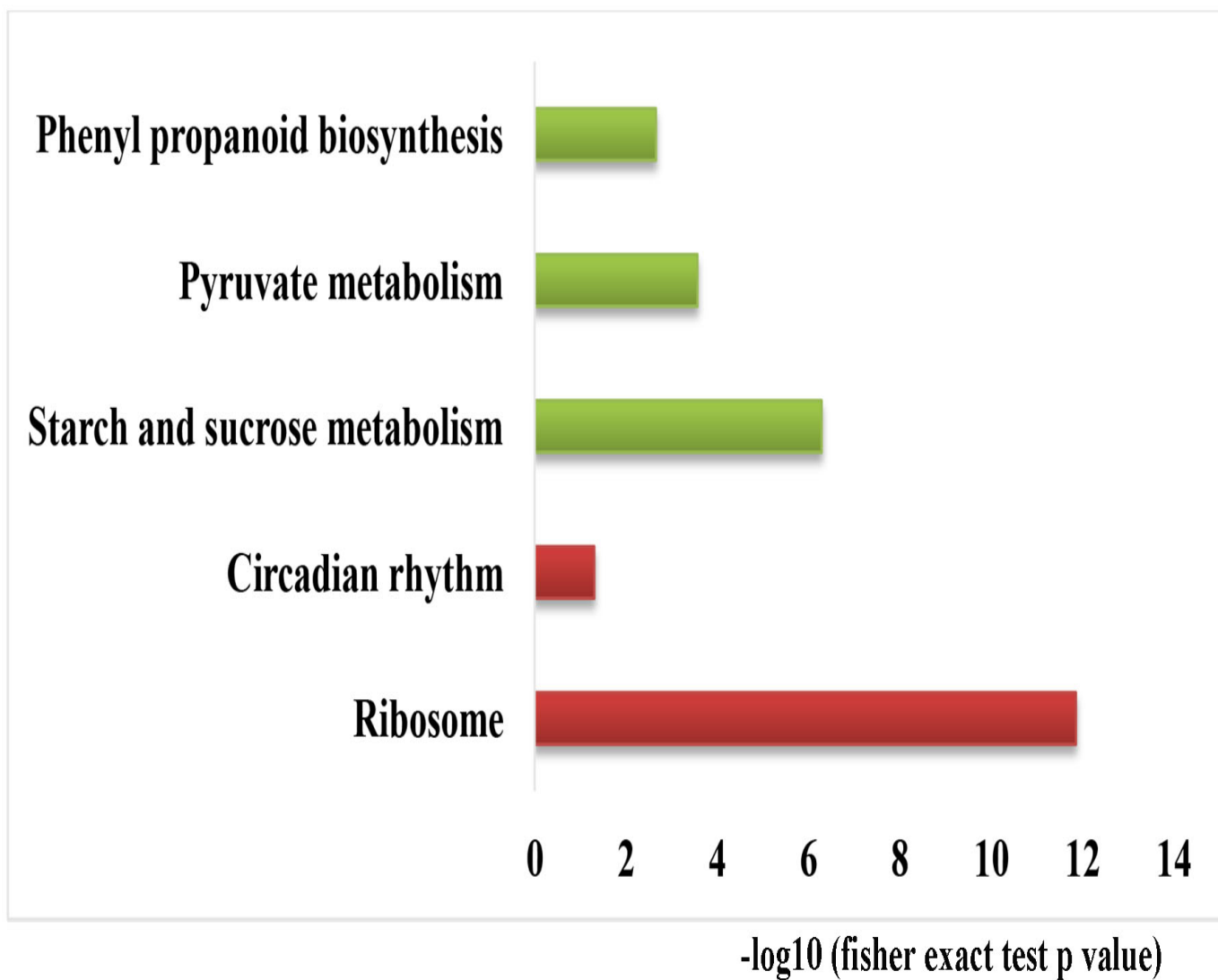

Supplement: Supplementary file 1 [file ijms-20-03528-s001.zip › ijms-535128-supplementary/Supplementary data/Figure S2.pdf]

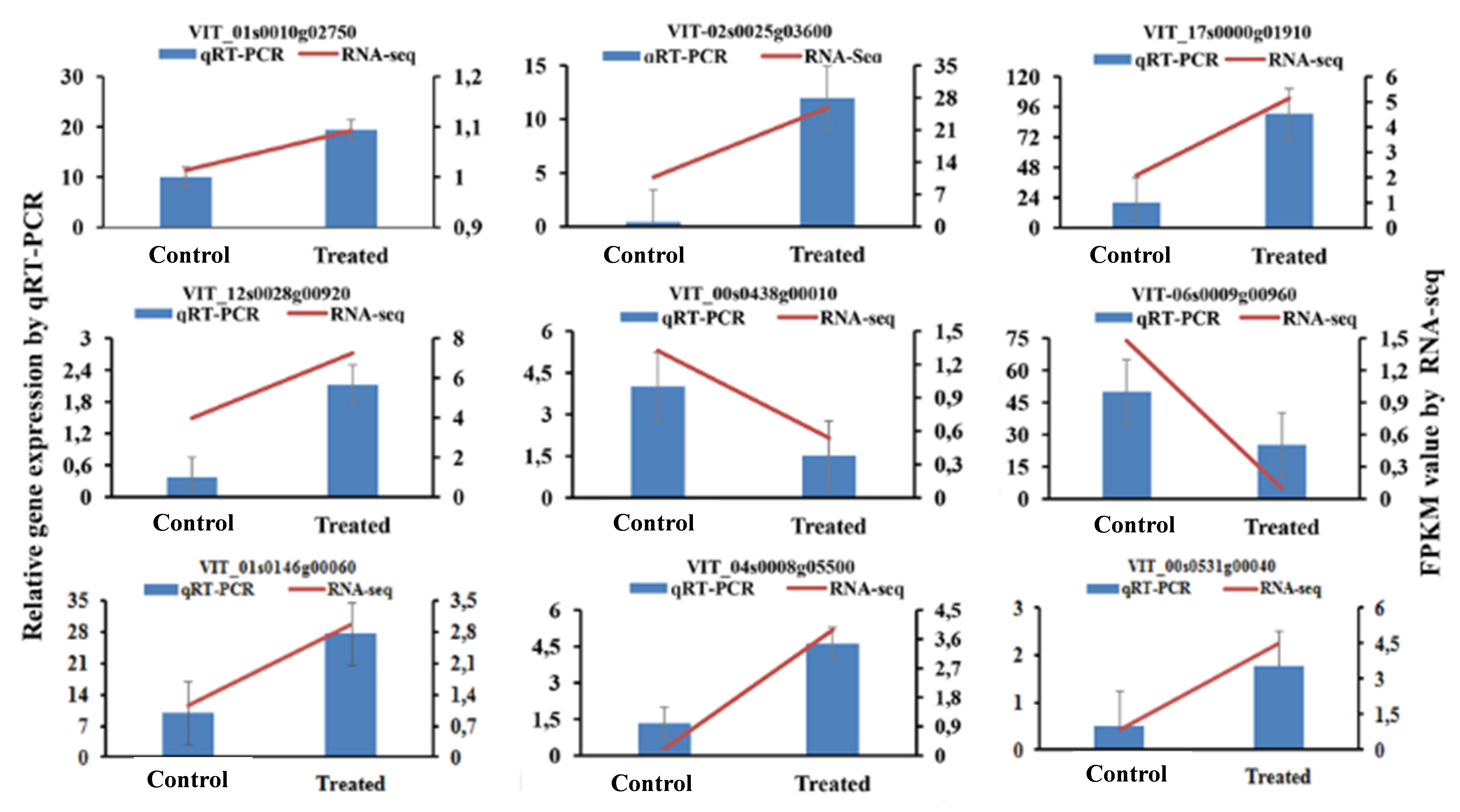

Supplement: Supplementary file 1 [file ijms-20-03528-s001.zip › ijms-535128-supplementary/Supplementary data/Figure S3.tif]

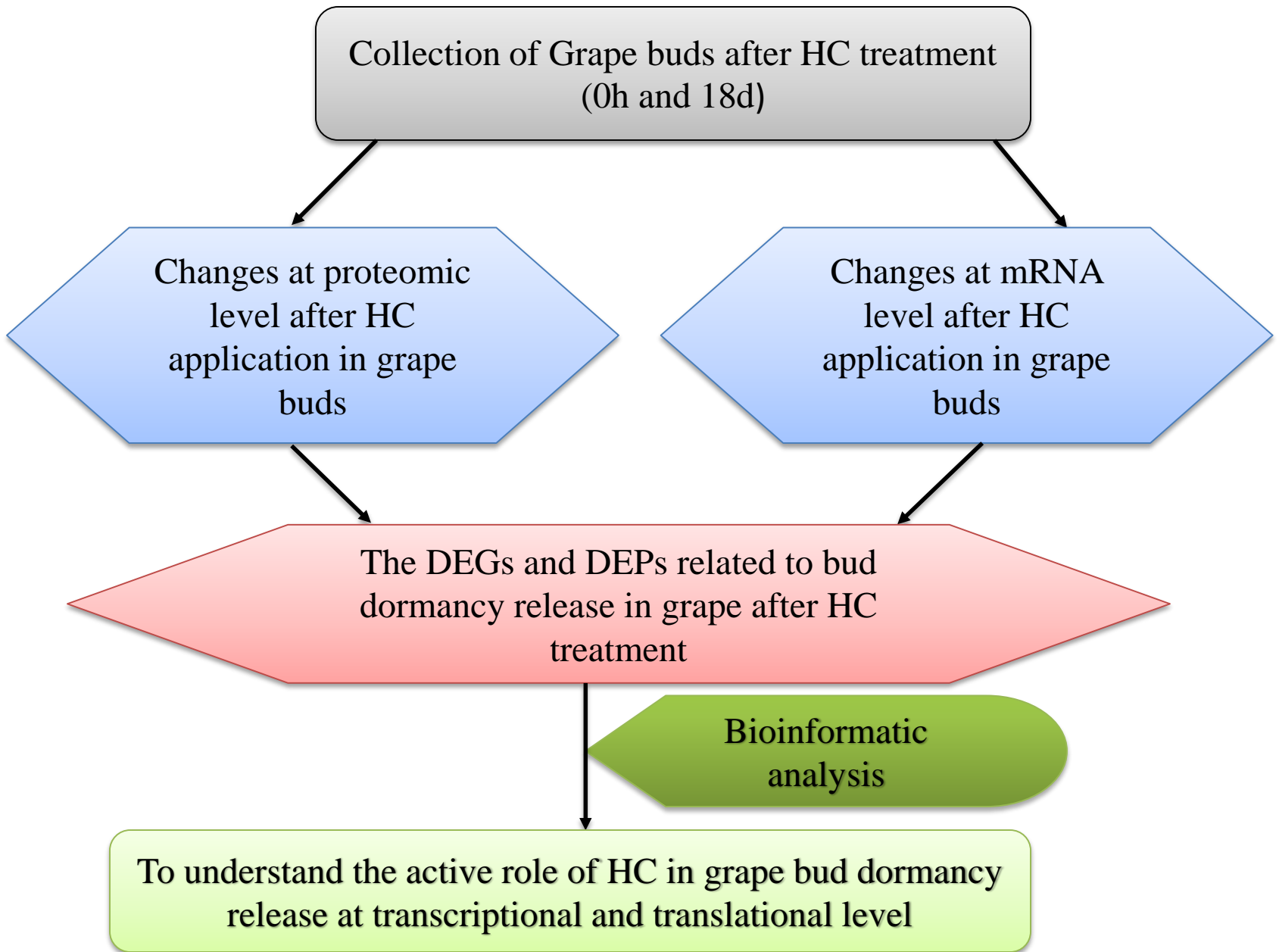

Supplement: Supplementary file 1 [file ijms-20-03528-s001.zip › ijms-535128-supplementary/Supplementary data/Figure S4.pdf]

**A**

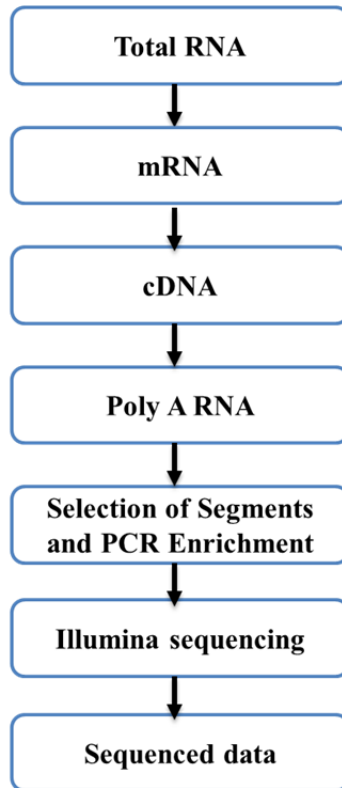

**B**

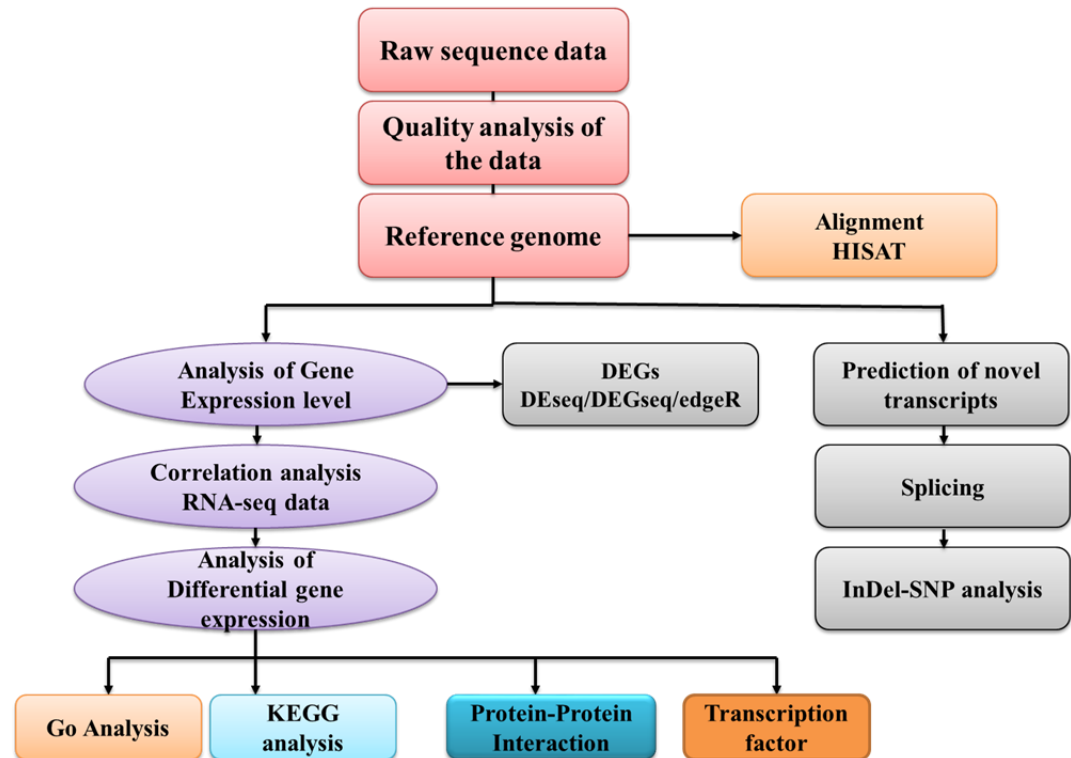

Supplement: Supplementary file 1 [file ijms-20-03528-s001.zip › ijms-535128-supplementary/Supplementary data/Figure S5.pdf]
